# Explainable AI for Depression Detection and Severity Classification From Activity Data: Development and Evaluation Study of an Interpretable Framework

**DOI:** 10.2196/72038

**Published:** 2025-09-11

**Authors:** Iftikhar Ahmed, Anushree Brahmacharimayum, Raja Hashim Ali, Talha Ali Khan, Muhammad Ovais Ahmad

**Affiliations:** 1 Department of Software Engineering, University of Europe for Applied Sciences, Germany, Potsdam, Germany; 2 Department of Mathematics and Computer Science, Karlstad University, Universitetsgatan 2, Karlstad, 65188, Sweden, 46 76-113 22 49

**Keywords:** artificial intelligence, explainable AI, depression, mental health, machine learning, activity data

## Abstract

**Background:**

Depression is one of the most prevalent mental health disorders globally, affecting approximately 280 million people and frequently going undiagnosed or misdiagnosed. The growing ubiquity of wearable devices enables continuous monitoring of activity levels, providing a new avenue for data-driven detection and severity assessment of depression. However, existing machine learning models often exhibit lower performance when distinguishing overlapping subtypes of depression and frequently lack explainability, an essential component for clinical acceptance.

**Objective:**

This study aimed to develop and evaluate an interpretable machine learning framework for detecting depression and classifying its severity using wearable-actigraphy data, while addressing common challenges such as imbalanced datasets and limited model transparency.

**Methods:**

We used the Depresjon dataset and applied Adaptive Synthetic Sampling (ADASYN) to mitigate class imbalance. We extracted multiple statistical features (eg, power spectral density mean and autocorrelation) and demographic attributes (eg, age) from the raw activity data. Five machine learning algorithms (logistic regression, support vector machines, random forest, XGBoost, and neural networks) were assessed via accuracy, precision, recall, *F*_1_-score, specificity, and Matthew correlation constant. We further used Shapley Additive Explanations (SHAP) and Local Interpretable Model-agnostic Explanations (LIME) to elucidate prediction drivers.

**Results:**

XGBoost achieved the highest overall accuracy of 84.94% for binary classification and 85.91% for multiclass severity. SHAP and LIME revealed power spectral density mean, age, and autocorrelation as top predictors, highlighting circadian disruptions’ role in depression.

**Conclusions:**

Our interpretable framework reliably identifies depressed versus nondepressed individuals and differentiates mild from moderate depression. The inclusion of SHAP and LIME provides transparent, clinically meaningful insights, emphasizing the potential of explainable artificial intelligence to enhance early detection and intervention strategies in mental health care.

## Introduction

Mental disorder is a global phenomenon with over a billion individuals suffering from various types of mental disorders. Mental health disorder usually affects a person’s thinking, interpersonal relationships, and emotions. It results in serious consequences such as risk of other diseases and suicide if not identified and treated properly in time. Depression is one of the most prevalent mental health disorders that can negatively impact an individual’s daily functioning and well-being. Studies have shown an increase in the number of depression and anxiety disorders during and post-COVID–19 pandemic, affecting both males and female demographics [1,2]. Younger demographics are found to suffer more from mental disorders than older citizens [3]. It is estimated that approximately 280 million of the population is suffering from depression [4].

Depression is characterized by changes in one’s temper or a sudden hysterical attitude to any circumstances or situation. Depression is regarded as the second most common reason for death, and 0.8 million deaths are attributed to depression [5]. Depression is an emotional disorder that triggers a sense of desolation, pessimism, and anhedonia that affect a person’s life. This leads to the disruption of daily activities in all aspects of life, influencing others around them as well. The etiology of these types of mental illness results from environmental and genetic factors [6]. A depressive illness can be classified as mild, moderate, and severe by degree of severity [7]. It is challenging for the physicians to diagnose it in the early stages and is often misdiagnosed as conditions such as vitamin deficiency [8]. It has also been linked with the rise of risk in cancer [9] and cardiovascular ailment [10] which can potentially decrease the life span of an individual. Even though it has high prevalence, it is often left untreated in many of the cases [11].

Depression rating scale is a screening tool that comprises a questionnaire that is used for detecting the severity phases of depression [12]. Montgomery-Asberg Depression Rating Scale (MADRS) was designed by Marie Asberg [13]. It focuses on ten signs of depression, which include apparent sadness, reported sadness, inner tension, reduced sleep, reduced appetite, concentration difficulties, lassitude, inability to feel, pessimistic thoughts, and suicidal thoughts [14]. The participant answers the questions with a score of 0 to 6. It is categorized as: 0 to 6 for normal or absence of symptom, 7 to 19 for mild, 20 to 34 for moderate and 35 to 60 for severe depression [15].

Variability is also seen in treatment response. While a person may respond well to treatment, others with the same symptoms may not respond to the same treatment in the same manner. Thus, posing a threat to an individual’s life as prolonging its diagnosis and treatment can cause serious conditions [16]. The diagnosis of depression is very challenging, and it is often misdiagnosed as it often mimics other types of disorders [17]. One such example is it is often misdiagnosed as anxiety [18]. Therefore, early detection and diagnosis of depression is paramount.

Over the preceding years, the domain of artificial intelligence (AI) has been evolving, and it has shown success in a plethora of fields including health care [19]. Machine learning (ML, a sub-field of AI) can potentially predict whether a person has a mental disorder or not, and if there is a degree of inclination to suicide [20]. Therefore, implementation of ML may benefit mental illness domain by characterizing those who are vulnerable and thus help the practitioners in the early identification of mental health disorders including depression.

The existing ML-based models for depression detection face several challenges, such as their inability to accurately differentiate between various types of depression. More advanced and sophisticated techniques such as deep neural networks are often restricted in their usage due to their “black box” approach, thereby lacking transparency and explainability [19]. This lack of interpretability is a major hindrance in health care applications where understanding the decision-making process is crucial [19]. In the context of diagnosis and treatment of depression, transparency and explainability are even more important.

AI models for depression require huge amounts of data which capture behavioral, psychological, and physiological indicators for effective modeling, and thus accurate detection and differentiation of depressive states. However, the collection of such datasets is often not possible due to challenges and limitations of data-collection methods [21]. An alternative is to use wearable devices to collect continuous, real-time data on various aspects such as physical activity, sleep patterns, heart-rate variability, and many other physiological indicators [21].

A number of studies have been conducted using a variety of datasets and ML models for diagnosis of several types of mental disorders. Paul and Juliet [22] considered the prediction of mental health disorders problem based on a dataset of self-reported information by the patients. The authors used four ML algorithms, namely logistic regression, k-nearest neighbor, decision tree, and random forest (RF). RF is identified as the best performing algorithm achieving an accuracy of 81%. However, the authors did not discuss other performance measures such as recall, precision, and *F*_1_-score. Zhang et al [23] suggested an architecture that uses magnetoencephalography to classify posttraumatic stress disorder. The authors used support vector machine as the main classifier with a recursive RF for feature selection. The model with selective features achieved an area under the curve value up to 0.9. Mikolas et al [24] focused on the automatic diagnosis of attention deficit hyperactivity disorder and argued that although recent advancements in the domain of ML helped in binary classification problem of identification of attention deficit hyperactivity disorder and healthy individuals, the problem of differentiating among multiple psychiatric conditions remains. The authors considered a dataset of 229 anonymized participants and observed that the best performance is obtained using automated feature selections. The study has certain limitations, such as a higher number of missing values in certain columns and lack of out-of-sample testing.

Spulber et al [25] explored whether activity data collected via actigraphy and depression severity levels are correlated. The study involved individuals experiencing major depressive episodes who were not receiving antidepressant treatment. The authors trained models to predict the MADRS score from activity data using ML techniques, selecting models based on accuracy and precision. These models were ranked as best, intermediate, or worst based on root mean squared error values. External validation was also conducted using data from patients undergoing treatment. However, this external validation introduced potential biases due to differences in patient conditions and treatment status. Garcia-Ceja et al [26] compared 2 models, namely RF and deep neural network (DNN), to identify healthy and nonhealthy participants from psychomotor data using the Depresjon dataset, which contains actigraphy data of control and condition groups. Due to class imbalance, different sampling methods such as random oversampling and Synthetic Minority Oversampling Technique (SMOTE) were adopted and were then compared with baseline and no oversampling after extracting three statistical features. It was found that RF with SMOTE achieved a higher *F*_1_-score of 0.73.

Frogner et al [27] implemented 1D convolutional neural networks (CNNs) to detect depression using activity data. Models were trained with different time segments, with 48-hours segments found to be optimal, to perform 3 classification tasks. The first task classifies depressive groups, the second classifies levels of depression (no depression, mild depression, and moderate depression based on MADRS score ranges: 0-10, 11-19, and >20, respectively), and the last task predicts the MADRS score. The evaluation was conducted using leave-one-participant-out cross-validation combined with majority voting. An *F*_1_-score of 70% was achieved for the first task, 30% for the second, and a mean square error (MSE) of 4.0 for the last one. The model performed well in classifying nondepressed participants but struggled with mild and moderate depression levels. Jakobsen et al [28] examined whether objective metrics of activity data could enhance current diagnostic techniques for depression by analyzing activity patterns using ML models. Three statistical attributes (mean activity level, standard deviation, and proportion of zero activity) were derived for RF and DNN models. CNNs were trained using raw activity data represented as a 24 × 60 image matrix. DNN with SMOTE achieved an accuracy of 84%, a true positive rate of 82%, and a true negative rate of 84%. However, a significant amount of misclassifications was observed, particularly in groups with overlapping activity patterns, such as patients with agitation.

Espino-Salinas et al [29] used the Depresjon dataset to create a framework for depression classification. Preprocessing involved converting motor activity data into vectors and creating matrices for input into neural networks. A 2D-CNN and DNN were then used to classify depressive and nondepressive subjects. The 2D-CNN achieved an accuracy of 76% and an *F*_1_-score of 72%, while the DNN achieved an accuracy of 72% and an *F*_1_-score of 69%. The *F*_1_-score was selected as the primary metric due to the imbalanced dataset, as it better reflects the model’s ability to handle unequal class distributions. The 2D-CNN outperformed DNN by achieving a better balance between sensitivity and specificity, demonstrating its potential for depression classification. To maintain focus and address space limitations, we direct readers to [30-35] for further details.

Despite significant advancements in ML techniques for mental health diagnosis, several gaps remain. A recurring limitation across studies is the relatively lower performance in distinguishing overlapping or nuanced conditions, particularly for depression severity levels. Models perform well in binary classifications (such as distinguishing between depressed and nondepressed individuals), but struggle with multiclass classifications or subtle variations in conditions, such as mild versus moderate depression. Another critical gap is the lack of explainable artificial intelligence (XAI) techniques in these studies. Current ML models, including CNNs and DNNs, operate as black box approaches. The black box approach means that these techniques offer limited insight into their decision-making processes. This lack of interpretability reduces trust and limits the practical application in clinical settings, where understanding why a model makes a specific prediction is as crucial as the prediction itself.

In this work, we use the data collected from wearable devices to investigate the application of ML algorithms in depression detection and severity level classification tasks. We use Adaptive Synthetic Sampling Technique (ADASYN) for addressing imbalanced datasets. Further, feature engineering is performed to extract meaningful features from the activity data. For the binary (depression: yes or no) and multiclass (predicting severity levels) classifications, we implement logistic regression, support vector machines, RF, XGBoost, and neural networks. The performance of the models is evaluated using accuracy, precision, recall, *F*_1_-score, and Matthew correlation constant (MCC). To further contribute toward the explainability of the model, we used Shapley Additive Explanations (SHAP) and Local Interpretable Model-agnostic Explanations (LIME) methods to explain the decision-making process of the best-performing models.

## Methods

### Overview

**Figure 1.**
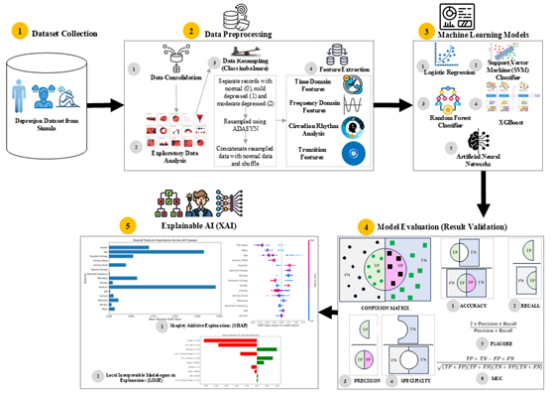
Graphical representation of methodology.

Figure 1 is a graphical representation of our methodology. We acquired data from the Depresjon dataset and conducted exploratory data analysis to understand the nature of the data. We observed a class imbalance, which is addressed using sampling techniques, followed by normalization of data. We then applied feature engineering to extract features from the data. This was followed by designing models for binary (depressed vs nondepressed) and multiclass (severity levels) classification tasks. After model implementation, we used accuracy, precision, recall, specificity, MCC, and *F*_1_-score as performance evaluation criteria. Finally, we applied the SHAP and LIME methods to explain the decision-making process of the selected model.

### Data Acquisition

The dataset adopted in this work is the “Depresjon” (Norwegian word for depression) dataset, available at Simula repository [36]. The dataset consists of motor activity of individuals recorded per minute for several days by using an actigraphy watch. The dataset consists of 2 parts: The first part comprises measurements of activity levels related to each individual in the condition and control group. The files consist of timestamp, date, and activity columns. The second part comprises the demographic information related to each participant. The dataset is enriched with several variables: an identification number (number), days with measurements (days), gender (1 for female and 2 for male), and age group of the participant (age). It also records the type of affective disorder (afftype). The presence of melancholia is noted with 1 for melancholia and 2 for lack of melancholia (melanch). The dataset differentiates between inpatients ‘1’ and outpatients ‘2’ (inpatient). Education level is categorized by years completed (education). Marital status is recorded as 1 for married or cohabiting and 2 for single (marriage). Work status is coded as 1 for working or studying and 2 for unemployed or sick leave or pension (work). Finally, the dataset includes MADRS scores before (madrs1) and after (madrs2) the inspection.

The original dataset comprised 2 folders (condition and control); it was important to merge the data into a single coherent set. The resultant dataset after the merge operation has 1440 data points. For binary classification (depressed vs nondepressed), the “condition” group is labeled as 1, and the “control” group as 0. For multiclass classification (assessing the severity levels of depression), the labels were assigned as follows: 0 for normal, 1 for mild depression, and 2 for moderate depression. Participants were categorized based on their MADRS score: 0‐6 for “Normal” (no depression), 7‐19 for “Mild” depression, and 20‐34 for “Moderate” depression. Note that although the standard MADRS scale includes a “Severe” category for scores >=35, our dataset contained no cases with scores in the severe range, so no “Severe” class was included.

### Addressing Data Imbalance

We observed that the data is imbalanced, which means that some output classes are more prevalent than others. Imbalance data can skew the performance of ML algorithms and require redressal. We used ADASYN for class imbalance problems. The intuition behind ADASYN is the usage of a weighted distribution for the less frequent instances in accordance with their difficulty of learning [37]. For the working of ADASYN, we refer the reader to He et al [37].

### Feature Engineering

The dataset consists of activity measurements recorded by an actigraph watch, resulting in a continuous stream of data collected at regular intervals for each patient. However, using these raw time series values is not an effective approach, as it can lead to an unnecessarily large number of interconnected features and fails to capture the nuanced behaviors and physiological rhythms indicative of mental health status. In the context of depression and related mood disorders, subtle variations in daily activity patterns, energy distribution across frequencies, and nonlinear fluctuations are particularly revealing. To address this, we summarized the continuous time-series data into key statistical features, thereby translating raw sensor data into indicators more closely tied to psychological and clinical insights. This feature engineering approach is a standard mechanism for managing continuous data streams while preserving essential patterns and variability [38], effectively reducing data dimensionality and computational requirements.

To capture the multifaceted nature of human activity and its potential connections to depressive symptoms, we systematically extracted features from 5 broad categories: (1) time domain, (2) frequency domain, (3) circadian rhythm analysis, and (4) transition features. Each category addresses different aspects of activity that can reflect mood changes or disruptions in daily routines often associated with mental health conditions.

### Time-Domain Features

Time-domain features describe statistical properties and variability in the activity signal. Disruptions or heightened variability in these measures can be linked to changes in energy levels and daily routines often observed in individuals experiencing mood disturbances. Time-domain features (such as mean activity, variability, higher-order moments, autocorrelation, and entropy) have long anchored actigraphy research. Historic studies show these measures consistently distinguish depressive from nondepressive motor patterns, providing a compact, behaviorally interpretable window on psychomotor change [25-27,35,38].

The following time domain features are extracted from the continuous stream of data.

- Mean SD: summarize central tendency and overall variability of the recorded activity levels. Marked fluctuations might indicate irregular sleep-wake cycles or energy instabilities common in depressive episodes.
- Skewness and kurtosis: capture the asymmetry and the “peakedness” of the activity distribution. Drastic changes in these metrics could signify periods of lethargy or bursts of hyperactivity, reflecting possible mood state shifts.
- IQR: Measures the spread between the 25th and 75th percentiles, providing a robust indication of how condensed or spread out the daily activity is, which might correlate with mood variability.
- Autocorrelation (AutoCorr): examines how similar activity levels are to themselves at varying time lags, helping detect repetitive patterns or circadian disruptions often seen in mental health disorders.
- Entropy: assesses the randomness or irregularity in activity patterns. High entropy might suggest erratic daily schedules, potentially symptomatic of poor sleep hygiene or mood instability.

### Frequency-Domain Features

Analyzing the distribution of activity power across different frequencies can illuminate underlying periodicities. Alterations in these periodic components often correspond to disrupted sleep-wake cycles or abnormal energy distribution, which are common in depression. Multiple actigraphy studies link these spectral abnormalities to symptom severity, confirming their diagnostic value for mood inference [25,29,35,38].

The features extracted are:

- Power spectral density (mean): a measure of how the signal’s power is spread across frequencies, reflecting the overall strength of periodic components in daily activity [39].
- Dominant frequency: identifies the frequency at which activity is most pronounced, commonly associated with daily (circadian) cycles. Deviations may mirror disrupted or shifted circadian rhythms in individuals with depressive symptoms.
- Spectral entropy: quantifies how “spread out” or concentrated the activity power is across frequencies. Greater spread could imply less stable routines and more variability in daily patterns, potentially linked to mood fluctuations.

### Circadian Rhythm Analysis

Mood disorders are often accompanied by disruptions in circadian rhythms. Examining the timing of key transitions can yield valuable clues about sleep-wake irregularities [40] (Activity Onset and Offset: identify the start and end of the primary daily activity period. Shifts in these values can reveal delayed or advanced phases of sleep, which are frequently observed in individuals with depression or other affective disorders).

### Transition Features

Activity does not always change smoothly throughout the day; abrupt transitions or steady patterns can be particularly telling of mental health status. Transition entropy captures the uncertainty or unpredictability inherent in the sequence of activity state transitions [41]. Transition entropy quantifies the randomness in the transitions between different activity states by evaluating the distribution of transition probabilities. Elevated transition entropy may reflect instability in daily routines or mood fluctuations, which are often observed in individuals with certain mental health conditions.

**Figure 2.**
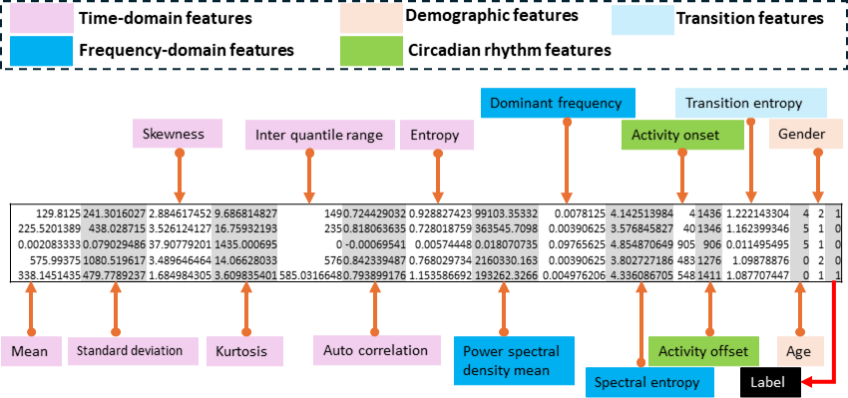
Snap of data after feature engineering.

Alongside the time- and frequency-domain metrics, we also tested a set of nonlinear descriptors (notably Fractal Dimension and the Hurst Exponent) to better capture the self-similar structure and long-range dependencies often observed in human activity signals. We implemented several published algorithms and tuned their parameters across multiple window lengths. Despite these efforts, the extraction pipeline consistently produced a large fraction of undefined or zero values, signaling numerical instability with our data resolution. After troubleshooting and sensitivity checks, the issue persisted and threatened to compromise both model robustness and interpretability. To safeguard the overall validity of the study, we therefore documented the attempted nonlinear analyses and excluded these features from the final feature set. Figure 2 is a pictorial representation of the dataset after feature engineering.

After feature engineering, we performed data normalization using *z*-score normalization technique as shown in Eq. (1).


$$z_{i}=\frac{x_{i}-\mu }{\sigma }$$

Note that $x_{i}$ and $z_{i}$ represent the individual feature score before and after standardization, whereas μ and σ represents the mean and standard deviation of the activity level scores.

#### Logistic Regression

Logistic Regression (LR) is a widely used supervised learning algorithm primarily employed for classification tasks [42]. LR estimates the probability that a given input belongs to a particular class by applying the logistic (sigmoid) function to a linear combination of input features. LR is favored for its simplicity, interpretability, and efficiency, especially when the relationship between the features and the target variable is approximately linear. It also provides probabilistic insights, allowing for the assessment of confidence in predictions. In addition, LR can be extended to multiclass classification problems using strategies such as One-vs-Rest (OvR) or multinomial LR [43]. Despite its simplicity, LR serves as a strong baseline model and is effective in scenarios where the decision boundary is linear or nearly linear.

#### Support Vector Machines

Support vector machines (SVM) are a popular model notably for classification problems. The core concept of SVM is to identify an optimal hyperplane that separates or distinguishes data points into different classes in an n-dimensional space [44]. For binary classification problems, SVM finds the optimal hyperplane that separates the 2 classes in the feature space. For the multiclass problem, two approaches are typically used: OvR and One-vs-One (OvO). In OvR, a binary classifier is used for each class against all other classes, whereas in OvO, a binary classifier is trained for every possible pair of classes [44]. This means that for a k-class classification problem, OvO trains $\frac{N\cdot \left(N-1\right)}{2}$ binary classifiers.

#### Random Forest

RF is a supervised ensemble learning algorithm that integrates the results of multiple decision trees to produce a more precise outcome [45]. Unlike decision trees, random forest has the ability to minimize overfitting [45]. RF is based on the bagging method where multiple models are trained using the subsets of features that are randomly selected, and the resulting predictions are aggregated either by voting or average mechanism. For regression, the prediction is the average prediction across all the decision trees, and for classification, it is the majority vote class predicted across the decision trees [46].

#### XGBoost

XGBoost (Extreme gradient boosting) is a ML algorithm based on the Gradient Boosting framework [47]. The idea of gradient boosting is based on developing multiple weak learners (like decision trees) with the expectation to aggregate the result to obtain a final strong learning model [47]. XGBoost builds an ensemble of decision trees sequentially, where each new tree attempts to correct the errors of the previous ones. XGBoost is particularly useful in dealing with imbalanced datasets [48].

#### Neural Networks

Neural networks (NN) are a class of powerful, flexible ML models inspired by the human brain’s network of neurons. Comprising layers of interconnected nodes (neurons), neural networks are capable of modeling complex, nonlinear relationships between input features and target variables. Each neuron applies a weighted sum of its inputs followed by a nonlinear activation function, enabling the network to learn intricate patterns and representations from the data [49].

In the context of mental health analysis, neural networks can effectively capture the complex temporal and spatial dependencies present in actigraphy data, making them well-suited for tasks such as depression classification and severity assessment [50]. Their ability to automatically learn feature representations from raw data reduces the need for extensive feature engineering, although they typically require larger datasets and more computational resources compared to traditional algorithms. The hyper-parameter settings are reported in Multimedia Appendix 1.

The hyper-parameter settings are reported in Multimedia Appendix 1.

### Evaluation Metrics

**Figure 3.**
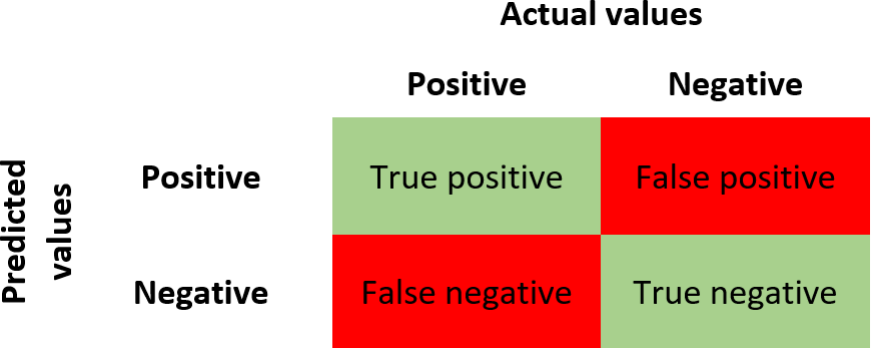
Confusion matrix.

In this study, we use accuracy, recall, specificity, precision, *F*_1_-score, and MCC as performance evaluation criteria for ML models. These performance evaluation measures are based on the confusion matrix, which represents a summary of accurate and misclassified instances for a classification problem. A graphical representation of the confusion matrix is given in Figure 3. A confusion matrix has 4 key elements called true positive (TP), false positive (FP), false negative (FN), and true negative (TN), defined as follows [51].

TP is an instance when the model classifies a positive instance correctly. FP is an instance when the model classifies a negative instance as a positive instance. FN is an instance when the model classifies a positive instance as a negative instance. TN is an instance when the model classifies a negative instance as a negative instance. Based on the definitions of various elements of the confusion matrix, in Table 1, we define our performance evaluation metrics.

**Table 1.** Performance metrics.

Metric	Formula
Accuracy (Acc)	$\frac{TP+TN}{TP+TN+FP+FN}$
Recall (Rec)	$\frac{TP}{TN+FN}$
Specificity (Spec)	$\frac{TN}{TN+FP}$
Precision (Prec)	$\frac{TP}{TP+FN}$
*F*_1_-score (F1)	$2\cdot \frac{Precision\times Recall}{Precision+Recall}$
MCC	$\frac{\left(TP\times TN\right)-\left(FP\times FN\right)}{\sqrt{\left(TP+FP\right)\left(TP+FN\right)\left(TN+FP\right)\left(TN+FN\right)}}$

## Results

### Performance Evaluation for Binary and Multiclass Classification Task

Table 2 highlights the performance of various ML models on the binary classification task. Among the evaluated models, XGBoost achieved the highest accuracy of 84.94%, significantly outperforming all other models. In contrast, LR was the least effective, with an accuracy of 70.68%. This ranking based on accuracy is consistently reflected across other metrics: XGBoost also leads in precision (83.40%), recall (87.45%), *F*_1_-score (85.29%), and MCC (0.7012), while LR scores the lowest in each of these categories. The remaining models, including SVM, RF, and neural networks, show a progressive improvement in performance, with SVM outperforming LR and RF and neural networks closely trailing XGBoost. This uniform trend across all evaluation metrics underscores the robustness of XGBoost as the superior model for this classification task.

**Table 2.** Performance comparison of various models.

Model	Accuracy	Precision	Recall	*F*_1_-score	Matthew correlation coefficient
Logistic regression	0.7068	0.6937	0.7471	0.7187	0.4156
SVM	0.7789	0.7648	0.8056	0.7842	0.5595
XGBoost	0.8494	0.8340	0.8745	0.8529	0.7012
Random forest	0.8301	0.8096	0.8663	0.8358	0.6639
Neural networks	0.8368	0.8275	0.8543	0.8397	0.6758

Furthermore, XGBoost exhibited an enhancement over the average performance of the other models across all evaluation metrics. Specifically, XGBoost surpassed the average accuracy, precision, recall, *F*_1_-score, and MCC by 7.2%, 6.42%, 6.83%, and 17.47%, respectively. This improvement underscores XGBoost’s superior capability in capturing complex patterns and interactions within the data, making it the most effective model in our study. The findings demonstrate the effectiveness of ensemble methods like XGBoost in outperforming traditional and other ML models, thereby offering enhanced predictive accuracy and reliability.

Table 3 presents the performance of various ML models on the multiclass classification task. XGBoost achieved the highest accuracy of 85.91%, along with precision, recall, and *F*_1_-scores exceeding 85%, and a MCC of 0.7895. In contrast, SVM and LR recorded the lowest accuracies of 53.22% and 54.12%, respectively, and underperformed across other metrics as well. RF and neural networks demonstrated strong performance with accuracies of 82.85% and 83.52%, respectively, and consistently higher precision, recall, *F*_1_-score, and MCC. These results indicate that XGBoost surpassed the average accuracy, precision, recall, *F*_1_-score, and MCC by 20.35%, 20.13%, 20.34%, 20.41%, and 33.07%, respectively.

**Table 3.** Performance comparison of various models for multi-class classification.

Model	Accuracy	Precision	Recall	F1	Matthew correlation constant
Logistic regression	0.5412	0.5414	0.5412	0.5386	0.3136
SVM	0.5322	0.5362	0.5322	0.5315	0.2999
XGBoost	0.8591	0.8602	0.8591	0.8581	0.7895
Random forest	0.8285	0.8308	0.8285	0.8276	0.7446
Neural networks	0.8352	0.8396	0.8352	0.8342	0.7554

### Statistical Significance Testing

To verify that the superior performance of XGBoost was not due to random chance, we conducted rigorous statistical significance testing, as detailed below. We ran a 10×3 repeated stratified cross-validation protocol (30 folds in total), preserving the original class distribution in every split. Within each fold, the model was fit to the training indices and its binary predictions on the held-out set were scored with the *F*_1_-metric, which is appropriate for moderately imbalanced problems. We then applied paired 2-tailed *t* tests between XGB and each rival model, followed by Holm correction to control the family-wise error rate at *α*=.05.

For binary classification, across the 30 cross-validation folds, XGB achieved the highest mean *F*_1_-score (0.851 ± 0.034) while its nearest competitor RF averaged 0.832; SVM, LR, and NN trailed at 0.659, 0.723, and 0.687, respectively. Paired significance testing confirmed that these differences are not due to sampling noise: the Holm-adjusted *P* values for XGB versus RF, LR, SVM, and NN were 3.6×10⁻⁴, 2.3×10⁻¹⁶, 2.7×10⁻¹⁷, and 1.7×10⁻²⁰, all well below 0.05, confirming that the superior performance of XGBoost is not by chance. Hence, XGB’s performance advantage is statistically significant against every other baseline in the study, supporting its selection as the primary model for downstream explainability analysis. The same trend was observed for multiclass classification.

## Discussion

### Principal Findings

In this section, we critically interpret XAI techniques (SHAP and LIME) to reveal the feature patterns that drive the depression-classification models, compare these explanations across XGBoost and neural-network architectures, and relate the findings to existing clinical evidence. One of the key critiques of ML models is their “black-box” nature [52]. ML models evolved over the last decade to become more complex, resulting in improved performance by virtue of modeling intricate relationships among the input features. However, the increased complexity also meant lower interpretability and explainability powers. In many use cases such as health care, the lack of transparency in decision making is critical, and the reasoning behind a model’s outcome is crucial for trust, accountability, and decision-making [52]. A variety of techniques is proposed in XAI to enhance the explainability of complex ML models. SHAP is a type of XAI framework derived from the concept of coalition game where a prediction is regarded as the “payoff” and the worthiness of the feature is assumed as the “player” [53]. It calculates the Shapley values and explains the prediction by showing how the features influenced the output. The importance of each feature that influenced the effectiveness of the ML algorithm is calculated as given in Eq. (2).


$$\phi_{i}\left(f,x\right)=\sum_{S\subseteq N_{i}}\frac{\left|S\right|!\cdot \left(N-\left|S\right|-1\right)!}{\left|N\right|!}\left[f\left(x_{s\cup \left\{x_{i}\right\}}\right)-f\left(x_{s}\right)\right]$$

Where: f represents the prediction model, x is the input feature vector, N is the set of features, S is a subset of features excluding the feature i that is, feature under XAI analysis, $x_{s}$ is the feature vector with features in subset S replaced by baseline values and $x_{i}$ is the value of feature i in the input vector x. For XAI analysis, we restrict to the Xgboost model only as it was identified as the best performing model.

LIME is a popular framework in XAI that helps make ML predictions easier to understand. LIME explains a model’s predictions by simplifying it in the local context of a specific instance. It does this by slightly altering the input data around the instance and analyzing how these changes affect the model’s predictions. This process highlights which features are most influential in the local neighborhood. To determine the importance of each feature, LIME builds a simple, interpretable model (such as a linear regression model) based on weighted data points. These weights are assigned according to how close the altered data points are to the original instance.

### XAI for Binary Classification

**Figure 4.**
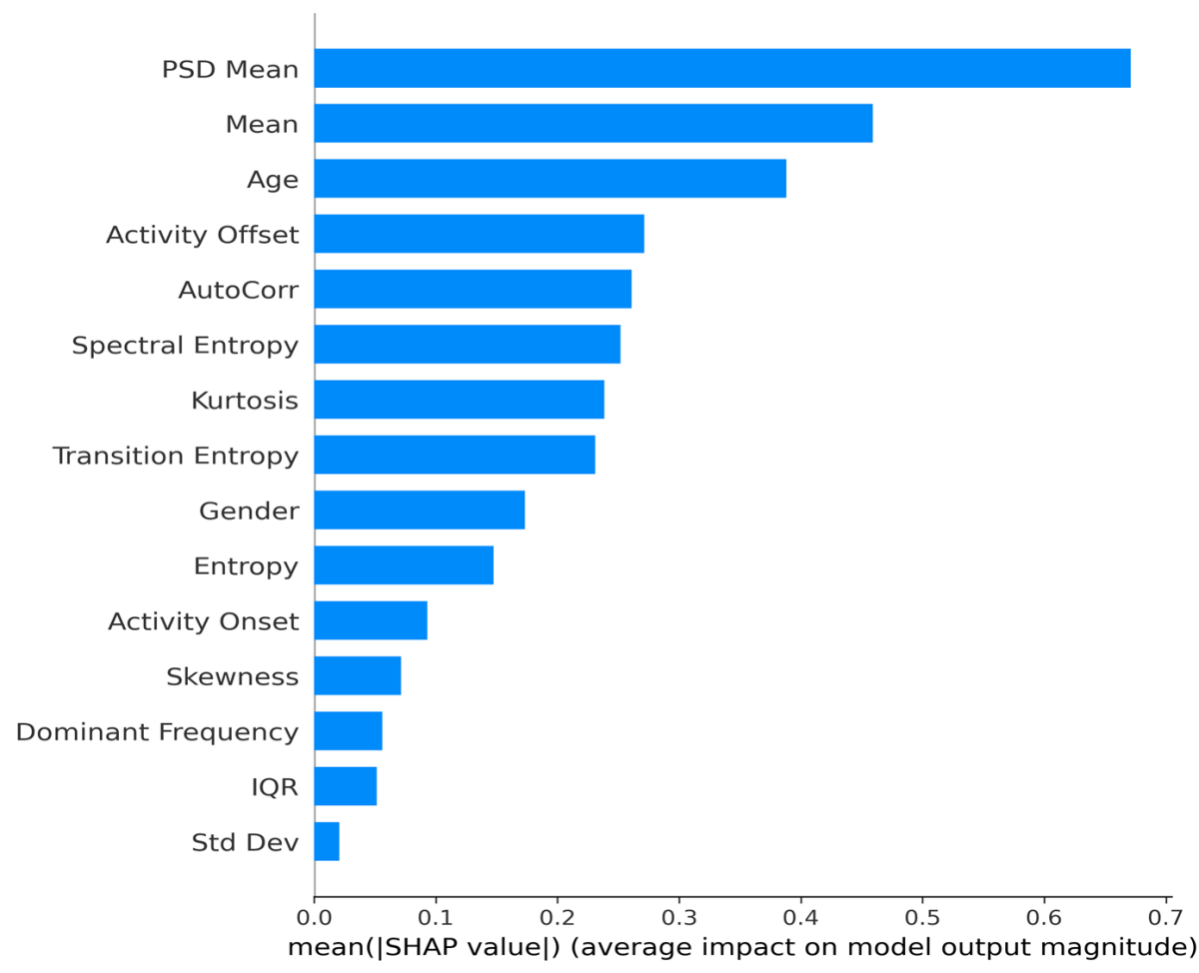
Binary classification feature importance.

**Figure 5.**
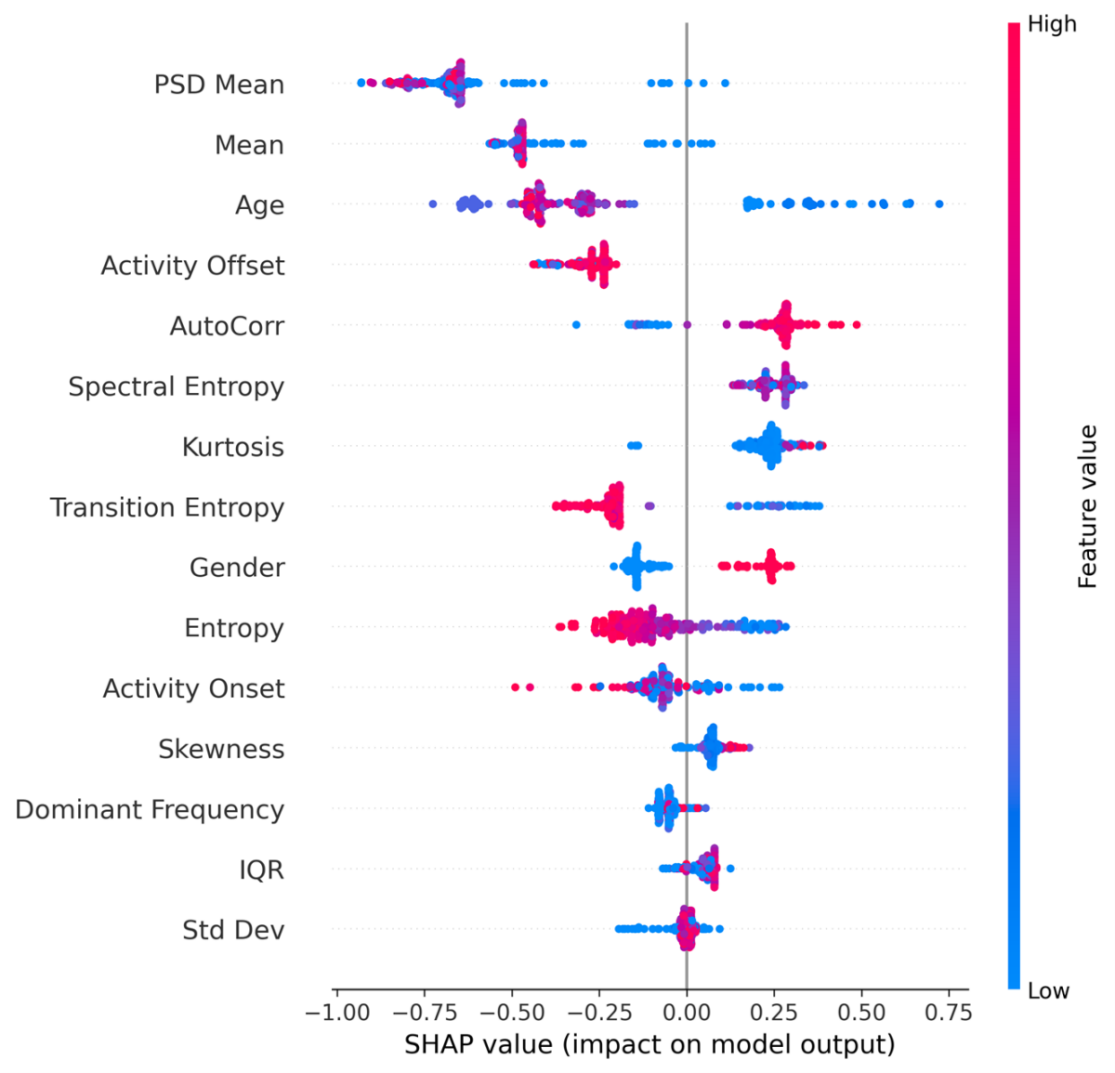
SHAPE summary plots for binary classification.

Two primary SHAP plots (Figures4  5) indicate how each feature affects the model’s decision boundary across the entire dataset. Figure 4 shows the average magnitude of SHAP values (mean absolute SHAP) per feature, highlighting the top predictors. Figure 5 depicts a dot summary where each point corresponds to a specific sample’s SHAP value. Points to the right (positive SHAP) push the classification toward “Depressed,” whereas points to the left (negative SHAP) favor “Non-Depressed.” Colors (red to blue) represent the actual feature value (eg, higher PSD Mean in red and lower PSD Mean in blue).

From these plots, we observe the following. PSD means consistently have the largest average impact. Larger values of the PSD mean (possibly reflecting a stronger low-frequency power in the activity data) correlate with a higher likelihood of depression. Mean activity level and age follow closely, indicating that demographic factors (eg, older individuals) and overall daily activity levels significantly shape the classification. Activity offset and AutoCorr also stand out, aligning with clinical findings that disrupted or rigid circadian rhythms (late offsets and high autocorrelation) are often associated with depressive behaviors.

PSD Mean consistently has the largest average impact. Larger values of the PSD mean (possibly reflecting a stronger low-frequency power in the activity data) correlate with a higher likelihood of depression.

Mean activity level and age follow closely, indicating that demographic factors (eg, older individuals) and overall daily activity levels significantly shape the classification.

**Figure 6.**
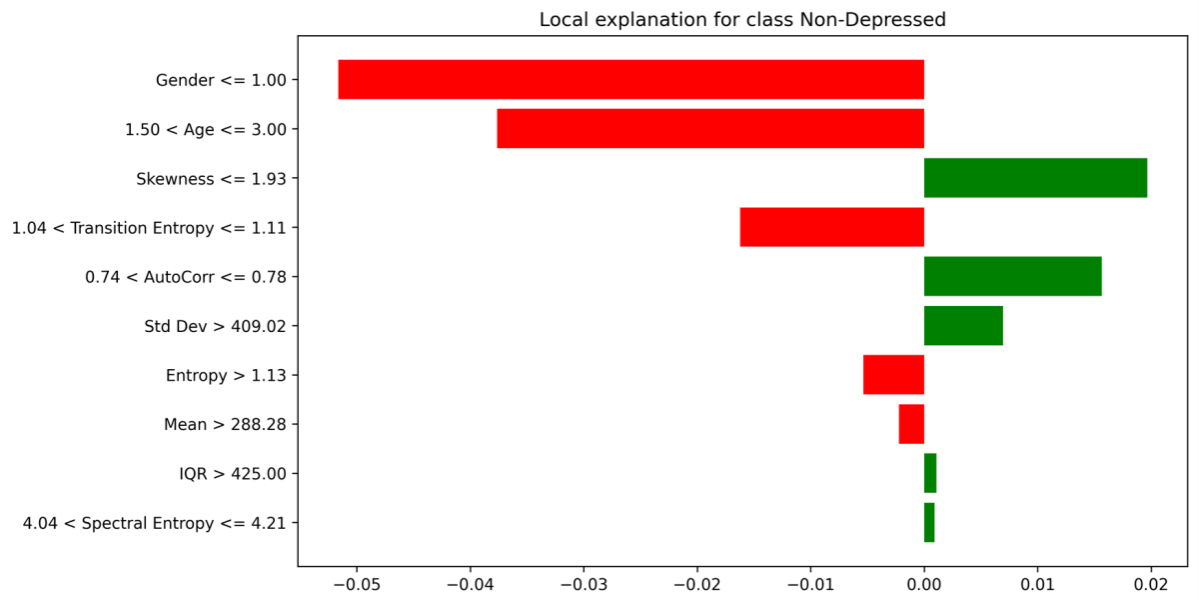
Local explanation for the non-depressed class.

We applied LIME to explain a single test instance in Figure 6. LIME creates locally linear “rules” indicating how small perturbations around this instance shift its predicted probability for depression. Features with red bars push the instance toward “Depressed,” while green bars push it toward “Non-Depressed.” For example, a condition like “Gender≤1.0” (female) or older “Age” might increase the model’s belief in depression if other activity-related features also align. LIME reported numeric weights for each condition, revealing how thresholds in AutoCorr, Entropy, or Skewness shape local decision boundaries. These rules coincide well with the SHAP findings (eg, higher autocorrelation frequently signals depressive risk).

While SHAP and LIME are theoretically different (SHAP uses game-theoretic Shapley values, and LIME uses local linear approximations), both converge on similar high-impact features, boosting confidence in the model’s learned representations. Specifically, PSD mean, mean activity level, and daily rhythm indicators (like activity offset, AutoCorr) repeatedly appear among the strongest predictors.

### XAI for Multiclass Classification

**Figure 7.**
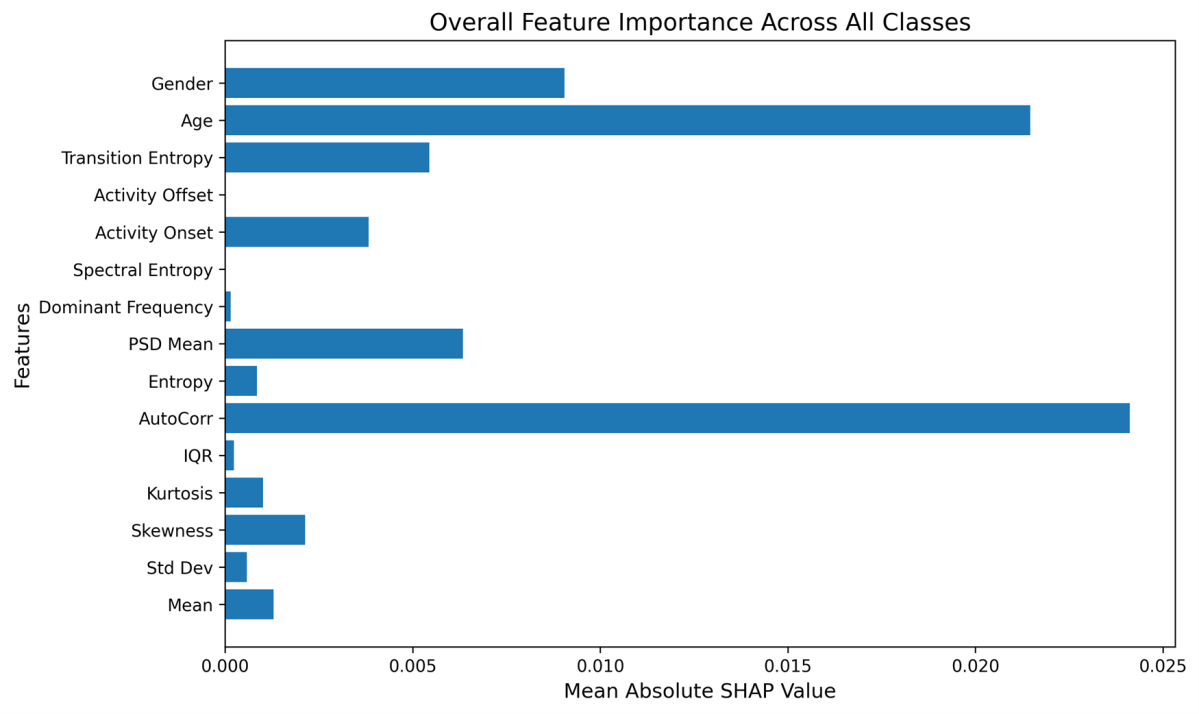
Multi-class feature importance.

We extended our explainable AI approach to a 3-class setting, distinguishing among “Normal Case,” “Mild Depression,” and “Moderate Depression.” SHAP was used for global insights (per-class and overall feature importance), while LIME was used for local (instance-level) explanations. Figure 7 displays the mean absolute SHAP values aggregated in all 3 classes, highlighting key characteristics that consistently influence the predictions of the model. Notably, AutoCorr and age emerge as dominant factors, aligning with clinical observations that older age groups, coupled with repetitive or rigid activity patterns, may exhibit a higher risk of depression.

**Figure 8.**
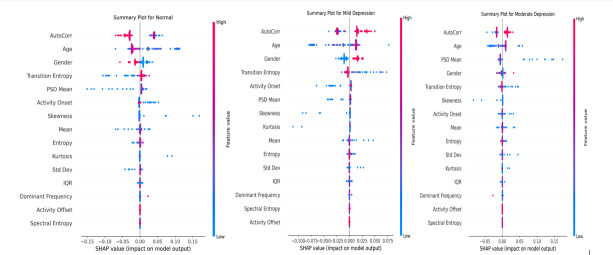
SHAP summary plots for multi-class classification.

For a more in-depth analysis of each class, we generated separate SHAP summary plots. Normal (Figure 8A): Lower AutoCorr typically favors the normal class, suggesting that a more flexible or varied daily rhythm (less repetitive activity) aligns with nondepressed status. Moderate values of age and PSD mean also push predictions away from mild or moderate depression.

Mild depression (Figure 8B): Mild cases show strong contributions from AutoCorr and transition entropy. In many instances, moderate daily rhythms—coupled with subtle changes in the frequency composition of activity (as captured by PSD Mean)—tilt the model toward mild depression. Moderate depression (Figure 8C): This class often manifests higher or more entrenched irregularities. Age, AutoCorr, and an elevated PSD mean appear to push an instance from mild toward moderate severity. Here, demographic and circadian disruptions intertwine more intensively, reflecting a deeper depressive state.

Across all classes, AutoCorr consistently ranks among the most critical features. However, its specific influence varies. Moderate autocorrelation can lead a case from normal to mild, while higher autocorrelation plus advanced age often indicates moderate depression. These distinctions underscore how the same feature can have class-dependent impacts based on subtle variations in its actual range or interaction with other features.

**Figure 9.**
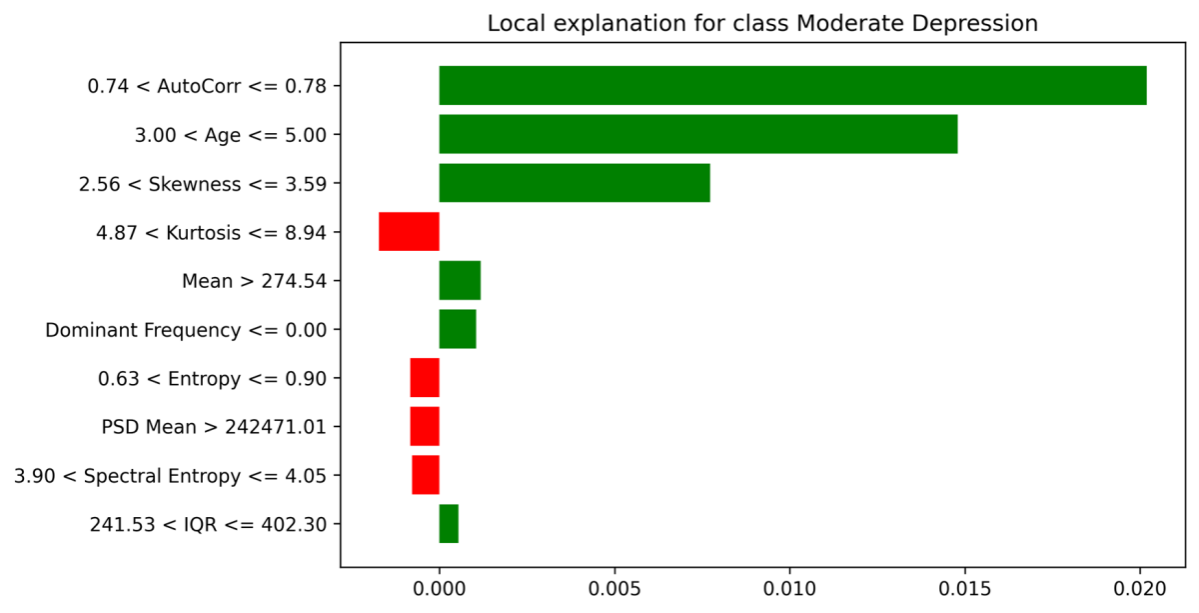
LIME explanation for the moderate depression class.

To validate SHAP’s per-class findings at the instance level, we used LIME for a single test example. Figure 9 focuses on the model’s explanation for the “Moderate Depression” class. LIME produces threshold-based “rules” (eg, “0.74<AutoCorr≤0.78”) that locally shift the probability of class membership. Green bars push the model toward selecting “Moderate Depression.” For instance, an *AutoCorr* range above 0.74 or age exceeding a certain threshold may substantially raise this class probability. Red bars pull the prediction away from “Moderate Depression,” indicating features or thresholds that conflict with the typical profile for moderate severity (eg, a lower PSD mean or higher Kurtosis).

This local view converges with the SHAP observations: circadian-related variables (eg, AutoCorr and Transition entropy) and demographic factors (eg, age) become pivotal. In mild cases, these metrics may appear only partially disrupted, whereas for moderate severity, they consistently register in ranges linked to stronger depressive indications.

### Comparing Explanations Across XGBoost and Neural Network Models

Although XGBoost delivered the highest accuracy, we repeated the XAI analysis for artificial-neural-network (ANN) to test whether the explanatory story was model-dependent. For binary classification, both models ranked PSD mean as the dominant driver of the depressed versus nondepressed decision, confirming the feature’s robustness. Beyond that, the explanations diverged: XGBoost distributed moderate importance across roughly ten additional variables (eg, mean, age, activity offset, and AutoCorr), whereas the ANN concentrated nearly all remaining attribution on just three features (activity onset, kurtosis, and activity offset). This comparison strengthens interpretability in 2 ways: the cross-model agreement on PSD mean validates it as a consistent physiological marker and the differing secondary patterns reveal architecture-specific sensitivities, offering complementary insights that would be missed if explanations were reported for XGBoost alone.

For binary classification, both models ranked PSD mean as the dominant driver of the depressed versus nondepressed decision, confirming the feature’s robustness. Beyond that, the explanations diverged: XGBoost distributed moderate importance across roughly ten additional variables (eg, mean, age, activity offset, and AutoCorr), whereas the ANN concentrated nearly all remaining attribution on just 3 features (activity onset, kurtosis, activity offset). This comparison strengthens interpretability in 2 ways: (1) the cross-model agreement on PSD mean validates it as a consistent physiological marker; and (2) the differing secondary patterns reveal architecture-specific sensitivities, offering complementary insights that would be missed if explanations were reported for XGBoost alone.

For multiclass classification, inspection of the mean-|SHAP| values reveals that XGBoost and the neural network rely on partly overlapping but ultimately different information in multiclass classification, and they do so on strikingly dissimilar attribution scales. XGBoost’s strongest signals come from temporal-structure and demographic variables—AutoCorr carries the largest impact, closely followed by age, with gender and transition entropy also substantial—whereas features such as activity offset contribute almost nothing. The ANN tells a nearly opposite story: its 2 most influential cues are the higher-order statistic kurtosis and activity offset, while the variables that dominate XGBoost shrink to the noise floor. Both models nevertheless converge on PSD mean as a relevant marker, which bolsters confidence in that spectral feature’s physiological validity even though its weight is modest in XGBoost and comparatively prominent in the ANN. It is also worth noting that the ANN’s absolute SHAP magnitudes are roughly 2 orders of magnitude smaller than the tree ensemble’s, reflecting the smoother decision surface learned by the network and the conservative estimates produced by KernelExplainer; consequently, comparisons should be made within each model’s scale rather than across scales. Taken together, the agreement on PSD mean and the divergence elsewhere show that the 2 architectures extract complementary patterns—trees capitalize on heterogeneous temporal-demographic interactions, whereas the network distills the data into distribution-shape metrics—providing a richer, cross-validated picture of how depression severity is encoded.

### Discussion of XAI Findings

The combined SHAP–LIME analysis reveals several consistent themes.

#### Circadian Rhythms and Activity Measures

Our model’s emphasis on autocorrelation and spectral activity features matches a large body of research linking disrupted daily rhythms to mood disorders. For example, clinical and experimental evidence shows that blunted or shifted circadian cycles are closely associated with depression [54,55]. A recent longitudinal study using wearable devices found that circadian phase shifts often precede worsening mood symptoms in depressed patients [56]. Furthermore, therapies that realign circadian timing (bright light therapy, social rhythm therapy, etc) reliably alleviate depressive symptoms [55], suggesting a causal role for rhythm disruption. That said, mood and circadian processes are bidirectionally linked [55,57], so altered activity patterns may both influence and reflect depressive states. In short, our findings are consistent with known associations; however, they do not by themselves prove that these rhythm-related features cause depression.

#### Age and Demographic Influence

Age and gender differences in depression risk are well documented [57,58], and our model captures these patterns. For instance, older adults tend to show earlier and less variable sleep–wake timing [59], which may interact with late-life depression risk. Epidemiological studies report that women have roughly twice the odds of major depression compared with men [58]; our model’s gender feature trends are in line with that. However, like other risk factors, age and gender effects are multifaceted and often indirect. Biological aging brings many changes (health, lifestyle, and brain function), and gender differences reflect hormonal, psychosocial, and diagnostic factors. Thus, while age and demographic features correlate with depression, the mechanistic pathways remain speculative. In practice, these variables likely modulate risk in complex, context-dependent ways [57], so caution is warranted in attributing causality to them without further study.

#### Distinction Between Mild and Moderate Depression

The multiclass SHAP plots show that certain transitions from mild to moderate often involve heightened daily-activity irregularities. The interplay of transition entropy and AutoCorr is particularly telling, resembling findings in psychological studies that link more chaotic sleep–wake transitions to deeper depressive states [60]. Nevertheless, these are still observational correlations. For example, it is unclear whether irregular sleep drives depression or mainly reflects its impact on physiology. Disentangling this requires targeted studies (eg, manipulating sleep schedules) beyond what SHAP and LIME alone can show.

#### Local Explanation Value

Per-instance force plots or LIME rules allow for clinically relevant breakdowns of risk factors, highlighting how subtle changes in demographic or circadian metrics can shift a patient’s classification from “Normal” to “Mild” or “Moderate Depression.” This local interpretability aligns with the literature’s emphasis on personalized medicine and the necessity of context-aware diagnostics [61].

In general, the XAI approach confirms that data-driven detection of depression is highly dependent on circadian and demographic characteristics—echoing established work on the connection between daily behavioral patterns and mental health status. By offering both a holistic (global) and case-specific (local) view, SHAP and LIME collectively bolster the transparency and reliability of ML in mental health applications. Note that although these associations are promising, confirming true causality will require targeted longitudinal and interventional studies beyond the correlations revealed by SHAP and LIME.

In this study, we presented a ML framework augmented with explainable AI for the detection and severity-level classification of depression based on wearable-actigraphy data. We demonstrated how XGBoost, coupled with ADASYN and feature selection, consistently achieved superior performance compared to other methods. The SHAP and LIME explanations revealed that circadian-related and demographic factors (eg, PSD mean, age, and AutoCorr) play central roles in identifying depressive states. By providing both global and local interpretability, the proposed system can help clinicians and researchers understand and trust the model’s decisions, supporting more targeted treatment plans and facilitating broader acceptance of AI-based tools in mental health care.

## Supplementary material

10.2196/72038 Multimedia Appendix 1 Hyperparameters of models.

## Abbreviations

ADASYN: Adaptive Synthetic Sampling Technique
AI: artificial intelligence
ANN: artificial-neural-network
CNN: convolutional neural networks
DNN: deep neural network
FN: false negative
FP: false positive
LIME: Interpretable Model-agnostic Explanations
LR: logistic regression
MADRS: Montgomery-Asberg Depression Rating Scale
MCC: Mathew correlation constant
ML: machine learning
MSE: mean square error
NN: neural networks
OvO: One-vs-One
OvR: One-vs-Rest
PSD: power spectral density
RF: random forest
SHAP: Shapley Additive Explanations
SMOTE: Synthetic Minority Oversampling Technique
SVM: support vector machines
TN: true negative
TP: true positive
XAI: explainable artificial intelligence
