# Supplementary material for: Explainable AI for Depression Detection and Severity Classification From Activity Data: Development and Evaluation Study of an Interpretable Framework
Source: JMIR Ment Health. 2025 Sep 11;12:e72038. doi: 10.2196/72038 (PMC12425426; doi:10.2196/72038)
Supplement: Multimedia Appendix 1 [file mental-v12-e72038-s001.docx]

**Appendix-A:**

Table A.1 : Hyper Parameters of Models for Binary Classification

| **Model** | **Key Hyper-parameters** |
| --- | --- |
| **Logistic Regression** | solver='liblinear', penalty='l2' (default), C=1.0 (default), max_iter=1000, random_state=42 |
| **SVM** | kernel='rbf', C=1.0, gamma='scale', probability=True, random_state=42 |
| **XGBoost** | n_estimators=150, max_depth=15, learning_rate=0.05, subsample=0.6, colsample_bytree=0.6, min_child_weight=2, gamma=0.05, alpha=1, objective='binary:logistic' (default), eval_metric='logloss', random_state=42 |
| **Random Forest** | n_estimators=22, max_depth=10, min_samples_split=5, min_samples_leaf=5, max_features=0.6, bootstrap=False, random_state=42 |
| **Neural Network** | Architecture: Dense(45, relu) → Dropout(0.15) → Dense(30, relu) → Dropout(0.10) → Dense(1, sigmoid); optimizer=Adam(learning_rate=0.01), loss='binary_crossentropy', epochs=75, batch_size=32 (default), weights seeded via random_state=42 |

Table A.2 : Hyper Parameters of Models for Binary Classification

| **Model** | **Key Hyper-parameters** |
| --- | --- |
| **Logistic Regression** | solver='lbfgs', multi_class='multinomial' (or ovr as stated), C=1.0 (default), max_iter=1000, random_state=42 |
| **SVM** | kernel='linear', C=1.0 (default), gamma='scale' (default for linear), probability=True, random_state=42 |
| **XGBoost** | n_estimators=100, max_depth=20, alpha=7, objective='multi:softmax', num_class=3, eval_metric='mlogloss', use_label_encoder=False, random_state=42 |
| **Random Forest** | n_estimators=22, max_depth=10, min_samples_split=5, min_samples_leaf=5, max_features=0.6, bootstrap=False, random_state=42 |
| **Neural Network** | Architecture: Dense(48, relu) → Dropout(0.20) → Dense(36, relu) → Dropout(0.20) → Dense(24, relu) → Dropout(0.20) → Dense(12, relu) → Dense(3, softmax); optimizer =Adam(learning_rate=0.005), loss = categorical_crossentropy |
